# Enhanced Grain Iron Levels in Rice Expressing an *IRON-REGULATED METAL TRANSPORTER, NICOTIANAMINE SYNTHASE*, and *FERRITIN* Gene Cassette

**DOI:** 10.3389/fpls.2017.00130

**Published:** 2017-02-07

**Authors:** Kulaporn Boonyaves, Ting-Ying Wu, Wilhelm Gruissem, Navreet K. Bhullar

**Affiliations:** Plant Biotechnology, Department of Biology, ETH ZurichZurich, Switzerland

**Keywords:** rice, iron biofortification, IRON-REGULATED METAL TRANSPORTER, NICOTIANAMINE SYNTHASE, FERRITIN

## Abstract

Micronutrient malnutrition is widespread, especially in poor populations across the globe, and iron deficiency anemia is one of the most prevalent forms of micronutrient deficiencies. Iron deficiency anemia has severe consequences for human health, working ability, and quality of life. Several interventions including iron supplementation and food fortification have been attempted and met with varied degrees of success. Rice, which is a staple food for over half of the world’s population, is an important target crop for iron biofortification. The genetic variability of iron content in the rice germplasm is very narrow, and thus, conventional breeding has not been successful in developing high iron rice varieties. Therefore, genetic engineering approaches have targeted at increasing iron uptake, translocation, and storage in the rice endosperm. We previously reported that *AtIRT1*, when expressed together with *AtNAS1* and *PvFERRITIN* (*PvFER*) in high-iron (NFP) rice, has a synergistic effect of further increasing the iron concentration of polished rice grains. We have now engineered rice expressing *AtIRT1, AtNAS1*, and *PvFER* as a single locus gene cassette and compared the resulting lines with transgenic lines expressing *AtIRT1* and *PvFER* gene cassettes. We also evaluated the efficacies of the *MsENOD12B* and native *AtIRT1* promoters for the expression of *AtIRT1* in rice in both types of gene cassettes, and found the native *AtIRT1* promoter to be a better choice for driving the *AtIRT1* expression in our biofortification strategy. All the single insertion transgenic lines have significant increases of iron concentration, both in polished and unpolished grains, but the concerted expression of *AtIRT1, AtNAS1*, and *PvFER* resulted to be a more effective strategy in achieving the highest iron increases of up to 10.46 μg/g dry weight. Furthermore, the transformed high iron lines grew better under iron deficiency growth conditions and also have significantly increased grain zinc concentration. Together, these rice lines have nutritionally relevant increases in polished grain iron and zinc concentration necessary to support human health.

## Introduction

Iron deficiency is one of the most prevalent micronutrient deficiencies, affecting around two billion people globally (WHO, 2016). Children and women in the developing countries are particularly vulnerable, with 300 million children and more than 500 million women suffering from iron deficiency anemia worldwide (WHO, 2015). Consequences of iron malnutrition include poor growth and mental development, lower cognitive ability in preschool children, and increased mortality of mother and child at birth (Beard and Connor, 2003; Stoltzfus et al., 2003). In order to control and prevent iron deficiency anemia, the WHO and UNICEF recommend intervention strategies including increased iron intake via dietary diversification, supplementation, and/or fortification, overall improved nutritional status by controlling other nutritional deficiencies as well as appropriate control of infectious diseases (WHO, 2001). However, these strategies are not always successful. Iron supplementation can cause increased severity of infectious diseases in the presence of malaria (Sazawal et al., 2006), while iron fortification of food is difficult since bioavailable iron compounds often cause changes in color and flavor (Hurrell, 2002). Biofortification of staple crops is therefore a recommended choice due to its sustainability and cost effectiveness. Rice is a staple food of more than half of the world’s population but contains only small amounts of micronutrients, including dietary iron. Furthermore, the micronutrient-rich layers of the grain, i.e., aleurone, bran, and husk are removed during polishing of the rice grain to increase its shelf life. Polished grains of widely grown rice varieties provide only around 2 μg/g of iron (Bouis et al., 2011) and therefore, rice is an important target crop for iron biofortification with recommended polished grain iron concentration of up to 15 μg/g dry weight (DW; Bouis et al., 2011).

Rice germplasm has a very narrow genetic variability for endosperm iron content (Gregorio et al., 2000; Glahn et al., 2002; Meng et al., 2005). Conventional breeding has therefore not been successful in increasing endosperm iron levels in popular rice varieties. Genetic engineering approaches are being used to biofortify rice, and these approaches rely on the information related to genetic network controlling iron uptake, transport, and storage. Generally, plants use reduction- (strategy I) or chelation- (strategy II) based mechanisms to acquire iron from soil. Strategy I, which is utilized by most higher plant species, involves the production of FERRIC CHELATE REDUCTASE (FRO) that reduces ferric (Fe^3+^) iron to ferrous (Fe^2+^) iron on root surface. Soluble Fe^2+^ is then transported into root cells by specific transporters, e.g., IRON-REGULATED METAL TRANSPORTER 1 (IRT1). IRT1 is a member of ZINC-REGULATED TRANSPORTER/IRON REGULATED TRANSPORTER (ZRT/IRT1)-RELATED PROTEIN (ZIP) transporter family (Eng et al., 1998). In contrast, graminaceous plants utilize strategy II and release phytosiderophores (PS) that form complexes with Fe^3+^, which are subsequently transported into roots by members of the YELLOW STRIPE (YS) and YELLOW STRIPE-LIKE (YSL) transporter families (Curie et al., 2001; Inoue et al., 2009). All PS belonging to the mugineic acid (MA) family are synthesized from *S*-adenosyl-L-methionine by a conserved pathway of three subsequent reactions catalyzed by NICOTIANAMINE SYNTHASE (NAS), NICOTIANAMINE AMINOTRANSFERASE (NAAT), and DEOXYMUGINEIC ACID SYNTHASE (DMAS) (Kobayashi and Nishizawa, 2012). Rice plants can acquire both Fe^2+^ and PS-Fe^3+^ utilizing strategy I and II, respectively (Ishimaru et al., 2006; Cheng et al., 2007), however, strategy I is only partially utilized because of non-inducible FERRIC CHELATE REDUCTASE activity in the root surface (Ishimaru et al., 2006).

Endosperm-specific expression of FERRITIN, which can store up to 4,500 iron molecules in its central cavity (Ford et al., 1984), has been the most successful approach for increasing iron storage in the rice endosperm. Rice *GLOBULIN* or *GLUTELIN* promoter-driven expression of *FERRITIN* could elevate iron concentration by 2- to 3.7-fold in the polished rice grains (Goto et al., 1999; Lucca et al., 2001; Vasconcelos et al., 2003; Qu le et al., 2005; Paul et al., 2012; Oliva et al., 2014). To improve overall iron uptake and translocation within the plants, strategies focusing on increased production of iron chelators and overexpression of certain intra- and intercellular transporters were tested (Masuda et al., 2008; Suzuki et al., 2008; Lee et al., 2009b, 2012; Ishimaru et al., 2010; Johnson et al., 2011; Zhang et al., 2012; Bashir et al., 2013; Trijatmiko et al., 2016). Constitutive overexpression of *NAS*, which is the most widely used gene among those related to the synthesis of PS, can increase iron levels two- to fourfold in polished rice grains (Masuda et al., 2009; Johnson et al., 2011; Lee et al., 2012). The effects of other MA biosynthesis genes and several of the YSL family transporters have also been tested in rice. Expression of IRON DEFICIENCY-SPECIFIC CLONE *3* (*IDS3*) from barley could elevate iron concentration by 1.4-fold in polished rice grains (Masuda et al., 2008; Suzuki et al., 2008). Transformation of rice with the barley *YS1* gene enhanced the iron concentration in leaves by 1.5-fold, however, no increase in grains was reported (Gomez-Galera et al., 2012). The overexpression of *OsYSL15* controlled by an *ACTIN* promoter in rice resulted in 1.2-fold higher iron concentration in the grains (Lee et al., 2009a). Transgenic rice expressing *OsYSL2* under the control of *SUCROSE TRANSPORTER* promoter had a fourfold iron increase in the endosperm (Ishimaru et al., 2010). Similarly, facilitating iron translocation from flag leaves to rice grains by decreasing the expression of the *VACUOLAR IRON TRANSPORTER* genes *OsVIT1* and *OsVIT2* resulted in a 1.8-fold iron increase in the endosperm (Zhang et al., 2012; Bashir et al., 2013). Among the regulatory factors of iron homeostasis, constitutive expression of the rice IRON-RELATED TRANSCRIPTION FACTOR 2 (OsIRO2), which regulates key genes involved in MA biosynthesis and iron-MA transport, resulted in a threefold increase of iron in rice grains when grown on calcareous soils (Ogo et al., 2011). It has also been reported that Zinc Finger protein 1 and 2 (OsHRZ1 and OsHRZ2), the negative regulators of rice iron deficiency responses, knockdown plants accumulate significantly higher iron in the shoots and grains, irrespective of soil iron conditions (Kobayashi et al., 2013).

Compared to the single gene strategies, the combination of genes required for effective iron transport and storage achieved higher increases in endosperm iron content. Endosperm-specific expression of *FERRITIN* (facilitating iron storage in endosperm) and *PHYTASE* (facilitating the release of phytic acid bound iron) together with the constitutive expression of *NAS* (NFP rice) resulted in a sixfold iron increase in polished grains (Wirth et al., 2009). Overexpression of barley *NAS1* (*HvNAS1*), *OsYSL2*, and soybean *FERRITIN* from a single construct increased iron content by 3.4- and 6-fold in polished grains of a Myanmar and a Japanese rice cultivar, respectively (Masuda et al., 2012; Aung et al., 2013). Similarly, overexpression of multiple genes involved in MA production, including *HvNAS1, HvNAAT*, and *IDS3*, together with the endosperm-specific expression of *FERRITIN* elevated endosperm iron content by fourfold (Masuda et al., 2013). Recently, the combination of *OsNAS2* and soybean *FERRITIN* expression resulted in iron levels reaching 15 μg/g in the polished rice grains (Trijatmiko et al., 2016).

The identification of rice *OsIRT1* and *OsIRT2* as well as their induced expression under iron deficiency conditions suggested an important function of IRT transporters in rice iron uptake (Ishimaru et al., 2006). This was further supported by the analysis of rice *naat* mutants that had impaired Fe^3+^-PS uptake but could grow normally with Fe^2+^ and under water-logged conditions (Cheng et al., 2007). The overexpression of *OsIRT1* in rice increased iron content in shoots, roots, and mature seeds, and improved tolerance to iron deficiency growth conditions (Lee and An, 2009). Expression of heterologous *IRT1* genes also improved rice growth under low iron conditions (Xiong et al., 2014) and increased iron content in mature grains (Tan et al., 2015). Recently, we found that expression of the *Arabidopsis thaliana IRT1* gene (*AtIRT1*) under control of the *Medicago sativa EARLY NODULIN 12B* (*MsENOD12B*) promoter increased iron levels 2.1-fold in polished grains (Boonyaves et al., 2016). We also transformed the high-iron NFP rice (Wirth et al., 2009) with the *MsENOD12B:AtIRT1* construct and achieved an additional 2.2-fold increase in endosperm iron content (Boonyaves et al., 2016). These results indicate that *AtIRT1* is useful for iron biofortification strategies when expressed in combination with other genes encoding iron transporters, chelators, and/or storage proteins.

We engineered rice expressing an *AtIRT1, AtNAS1*, and *PvFERRITIN* gene cassette and compared the resulting lines with transgenic lines expressing *AtIRT1* and *PvFERRITIN* gene cassettes. We also evaluated the efficacies of the *MsENOD12B* and native *AtIRT1* promoters for the expression of *AtIRT1* in rice in both types of gene cassettes. All transgenic lines show significant increases of iron content, both in the polished and unpolished grains, but highest iron levels of up to 10.46 μg/g DW were obtained in lines expressing the *AtIRT1, AtNAS1*, and *PvFERRITIN* gene cassette.

## Materials and Methods

### Construct Generation, Rice Transformation, and Plant Growth Conditions

**FIGURE 1:**
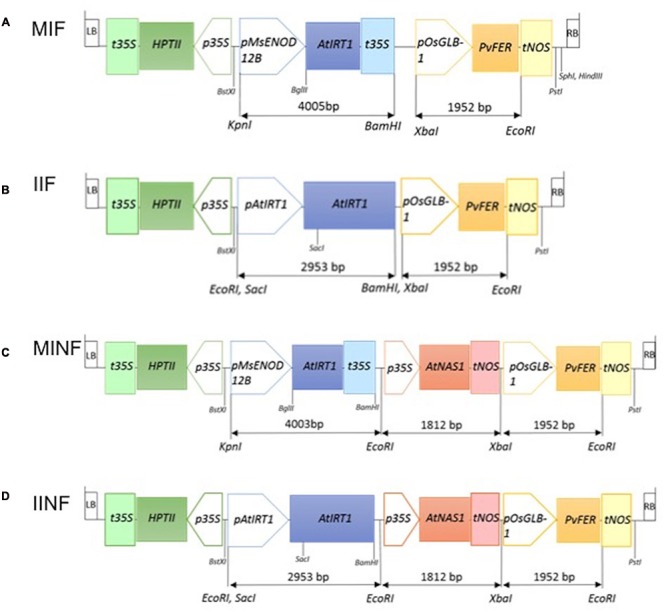
**Schematic illustration of four constructs used for transformation.** MIF **(A)**, IIF **(B)**, MINF **(C)**, and IINF **(D)** represent different transgene constructs (for details, see Materials and Methods). The length of the different DNA fragments is indicated in base pairs (bp). LB, T-DNA left border; RB, T-DNA right border; *t35S*, cauliflower mosaic virus (*CaMV*) *35S* terminator; *HPTII, HYGROMYCIN PHOSPHOTRANSFERASE* gene; *p35*S, *CaMV 35S* promoter; *pMsENOD12B, Medicago sativa EARLY NODULIN 12B* promoter; *AtIRT1*, Arabidopsis *IRON REGULATED METAL TRANSPORTER 1* gene; *pAtIRT1, AtIRT1* promoter; *AtNAS1*, Arabidopsis *NICOTIANAMINE SYNTHASE 1* gene; *tNOS, NOPALINE SYNTHASE* terminator; *pOsGLB-1*, rice *GLOBULIN-1* promoter; *PvFER, Phaseolus vulgaris FERRITIN* gene; *Bst*XI, *Kpn*I, *Eco*RI, *Sac*I, *Bgl*II, *Bam*HI, *Xba*I, and *Pst*I: restriction enzyme sites.

*MsENOD12B* or native *AtIRT1* promoter-driven expression of *AtIRT1* was combined with the endosperm-specific expression of *PvFERRITIN* (*PvFER*) under the control of rice *GLOBULIN-1* (*GLB-1*) promoter and the *CaMV35S* promoter-driven constitutive expression of *AtNAS1*. Four constructs with different combinations of these promoters and genes were generated, i.e., MIF (*pMsENOD12B::AtIRT1*, and *pOsGLB-1::PvFER*); IIF (*pAtIRT1::AtIRT1*, and *pOsGLB-1::PvFER*); MINF (*pMsENOD12B::AtIRT1, pCaMV35S::AtNAS1*, and *pOsGLB-1::PvFER*); and IINF (*pAtIRT1::AtIRT1, pCaMV35S::AtNAS1*, and *pOsGLB-1::PvFER*) (**Figure 1**). The *AtIRT1* promoter fused to the full-length *AtIRT1* cDNA sequence in the pCAMBIA1300 (pIRT1) plasmid was kindly provided by Prof. Dr. Mary Lou Guerinot. The DNA fragments containing *pCaMV35S::AtNAS1::tNOS* and *pGLB-1::PvFER::tNOS* were excised from the plasmid used for generating the NFP rice (Wirth et al., 2009) and inserted into *Bam*HI and *Pst*I site of pIRT1 to generate IIF and IINF constructs (**Figure 1**). In the case of MIF and MINF constructs, the plasmid pCAMBIA1300 was cut at the *Bam*HI and *Pst*I sites and the DNA fragments containing *pGLB-1::PvFER::tNOS* (pF), *pCaMV35S::AtNAS1::tNOS*, and *pGLB-1::PvFER::tNOS* (pNF) were appropriately inserted. The *AtIRT1* gene (1,374 bp) was amplified from pIRT1 by using forward primer 5′-AGATCTCACACAACAATCCAAAAG-3′ and reverse primer 5′-GTCGACGAAAAAGCAGCAAAAGTT-3′, followed by restriction digest of the *AtIRT1* fragment with *Bgl*II and *Sal*I before inserting it into pMSB containing the *MsENOD12B* promoter (Terada et al., 2001). The *MsENOD12B::AtIRT1::tCaMV35S* fragment was excised and then inserted into pF and pNF, respectively, to generate MIF and MINF constructs (**Figure 1**). These DNA constructs were transferred into *Oryza sativa* ssp. *japonica* cv. Nipponbare (NB) using transformation with *Agrobacterium tumefaciens* strain EHA105 (Hood et al., 1993). Transformation, selection, and regeneration were based on the protocol described by Nishimura et al. (2006). Putative transformants selected by hygromycin were screened for the presence of *AtIRT1, AtNAS1*, and *PvFER* by PCR using following primers: *AtIRT1*fw, 5′-TGATGCTACCTTGAAGCTTAG-3′ and *AtIRT1*rv, 5′-TCAACTGCGCCGGAAGAATG-3′; *AtNAS1*fw, 5′-ACTCGTGTCCACGTGCTTAC-3′ and *AtNAS1*rv, 5′- TTGTTCATGATCGCGTGAATC-3′; and *PvFER*fw, 5′- GCCTCAACTGTGCCTCTTACT-3′ and *PvFER*rv, 5′- CCACAACACTACAAGTTCTTAC-3′. Southern blot hybridization using digoxigenin (DIG) labeling was conducted on *Bam*HI digested genomic DNA isolated from the transgenic lines to select the lines with a single copy transgene insertion. *AtIRT1* or *AtNAS1* fragments amplified by specific primers as described above were used as probes to detect the transgenes. Single copy insertion lines were selected for each construct, i.e., 2 lines for MIF, 5 lines for IIF, 8 lines for MINF, and 13 lines for IINF (Supplementary Figure S1). Selected transgenic plants together with NB control were grown on commercial soil (Klasmann-Deilmann GmbH, Germany) in the greenhouse with 80% humidity/30°C/12 h light and 60% humidity/22°C/12 h dark. Phenotypic characteristics of the transgenic lines in T_1_ are presented in Supplementary Table S1. Quantification of divalent metals and transgene expression was conducted on plants in the T_2_ generation.

### Metal Ion Measurements

Grain samples were de-husked to obtain unpolished brown grains. The de-husked grains were milled with a grain polisher (Kett grain polisher “Pearlest,” Kett Electric Laboratory, Tokyo, Japan) for 1 min to obtain polished grains. To measure iron concentration in shoots and roots, 1-week-old seedlings were grown on hydroponic solution (Kobayashi et al., 2005) containing different iron supplies (5 μM Fe-EDTA for iron-deficient and 100 μM Fe-EDTA for iron-sufficient conditions) for 1 week. Shoot and root samples were dried at 60°C for 5 days. Ground samples were boiled in 15 ml of 65% v/v HNO_3_ solution at 120°C for 90 min. Subsequently, 3 ml of 30% v/v H_2_O_2_ were added and boiled at 120°C for 90 min. Metal concentrations were determined using inductively coupled plasma-optical emission spectroscopy (ICP-OES) (Varian Vista-MPX CCD Simultaneous ICP-OES). The wavelength used for iron, zinc, manganese, and copper were 238.204, 213.857, 257.610, and 324.754 nm, respectively. The National Institute of Standards and Technology (NIST) rice flour standard 1568a (NIST, USA)^1^ was treated and analyzed in the same manner and used as quality control for every measurement. Data were analyzed using Student’s *t*-test, and the statistically significant differences among the tested lines were determined based on *P* = 0.05 and *P* = 0.01.

### Pearl’s Prussian Blue Staining

Rice seeds were stained as described (Prom-u-Thai et al., 2003). Unpolished grains were cut longitudinally with a ceramic knife (Cerastar^®^, Germany) in a Petri dish. The cut grains were submerged in freshly prepared Pearls’ Prussian blue solution (2% hydrochloric acid and 2% potassium ferrocyanide) for 10 min and then washed gently in distilled water for 2 min. Stained seeds were examined by a stereomicroscope (Keyence, VHX-1000D).

### Quantitative Real-Time PCR

To determine transgene *AtIRT1* expression, rice T_2_ seedlings were germinated on hydroponic solution (Kobayashi et al., 2005) in a Petri dish sealed with a tape, particularly to improve collection of root hairs. Total RNA was extracted from shoots and roots of 5-day-old seedlings using Trizol^®^ reagent (Invitrogen, USA). To obtain total endosperm RNA, endosperm was excised from rice grains at 3 weeks after anthesis. Extraction buffer containing 0.15 M NaCl and 1% sarkosyl was added into the ground endosperm samples followed by purification with guanidine hydrochloride buffer (Sharma et al., 2003). The RNA was treated with DNase I (Thermo Fisher Scientific Inc., USA) to remove DNA contamination. First-strand cDNA was synthesized using the RevertAid^TM^ first strand cDNA synthesis kit (Thermo Fisher Scientific Inc., USA). Quantitative real-time PCR (qRT-PCR) was performed using the 7500 FAST Real Time PCR system (Applied Biosystem, Inc., USA). Total reaction volume of 20 μl included 4 μl cDNA, 0.5 μl forward primer, 0.5 μl reverse primer, 10 μl SYBR^®^ Green PCR Master Mix (Applied Biosystems Ltd., USA), and 5 μl H_2_O. The Ct value was calculated using the 7500 Fast System Software (Applied Biosystems, Inc., USA). Relative quantification of the transgene by 2^-ΔCt^ analysis was performed using a primer set as provided in Supplementary Table S2. The data were normalized in reference to the endogenous expression of *Os01g0147200* (IWS1, C-terminal family protein), which was selected using Genevestigator^®^^2^ based on its stable expression.

## Results

### Concerted Expression of *AtIRT1, AtNAS1*, and *PvFERRITIN* Increases Iron Concentration in Polished Rice Grains

**FIGURE 2:**
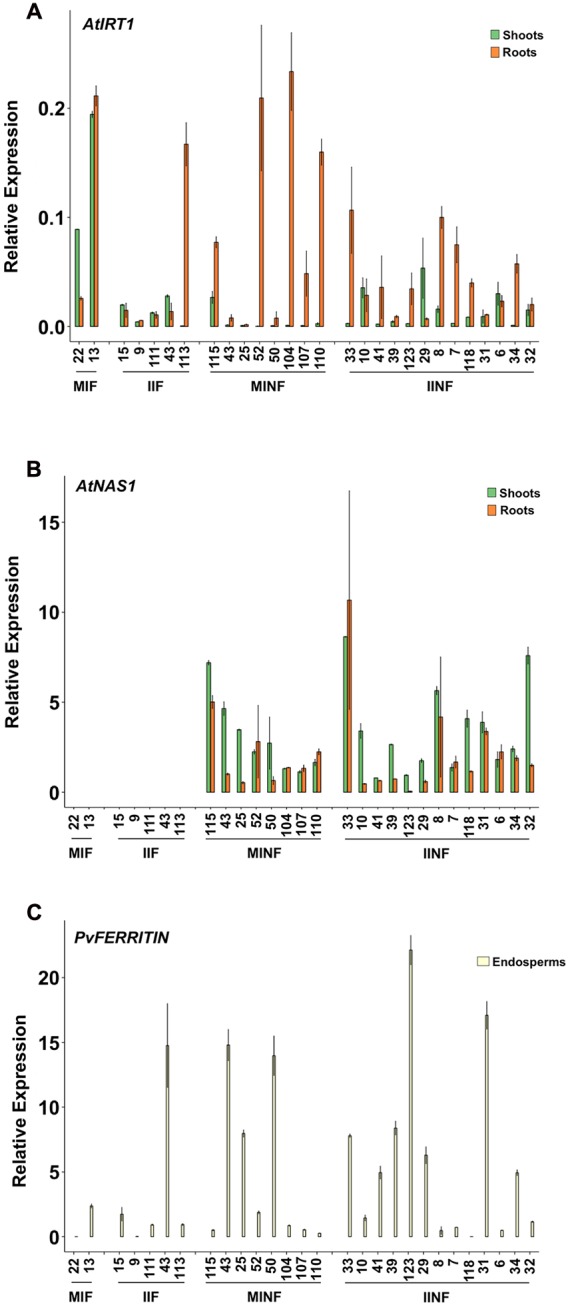
**Expression analysis of *AtIRT1, AtNAS1*, and *PvFERRITIN* in transgenic lines.** Relative expression of *AtIRT1* **(A)** and *AtNAS1* **(B)** in roots and shoots of T_2_ seedlings, and the relative expression of *PvFERRITIN* **(C)** in the endosperm of T_3_ grains was analyzed. Values are the average of three biological replicates (±standard deviation). MIF, plants with the *pMsENOD12B::AtIRT1::t35S* and *pGLB-1::PvFER::tNOS* transgenes; IIF, plants with the *pAtIRT1::AtIRT1* and *pGLB-1::PvFER::tNOS* transgenes; MINF, plants with the *pMsENOD12B::AtIRT1::t35S, pCaMV35S::AtNAS1::tNOS* and *pGLB-1::PvFER::tNOS* transgenes; IINF, plants with the *pAtIRT1::AtIRT1, pCaMV35S::AtNAS1::tNOS* and *pGLB-1::PvFER::tNOS* transgenes.

The expression of the transgenes was analyzed in several independent single copy insertion lines for each construct (**Figure 2**; see also **Figure 1** and Supplementary Figure S1). Shoots and roots generally showed the expected expression of *AtIRT1* and *AtNAS1* but not *PvFERRITIN*, consistent with the choice of promoters, although the activity of the promoters was variable among different independent lines (**Figure 2**). However, in most lines *AtIRT1* was more strongly expressed in roots, especially in the MINF and IINF lines. Both *MsENOD12B* and *AtIRT1* promoters are active in root hair and epidermal cells (Terada et al., 2001; Cailliatte et al., 2010), and it cannot be excluded that the *AtIRT1* expression specificities in **Figure 2** were biased by proportional differences in the amount of root hair cells in the collected root samples. In some lines *AtIRT1* was expressed more strongly under the control of the *MsENOD12B* promoter, but in general both *MsENOD12B* and *AtIRT1* promoters showed variable activities, most likely resulting from position effects on the transgene constructs. *PvFERRITIN* was expressed only in the rice endosperm, consistent with the endosperm specificity of the *OsGLB1* promoter (**Figure 2**).

**FIGURE 3:**
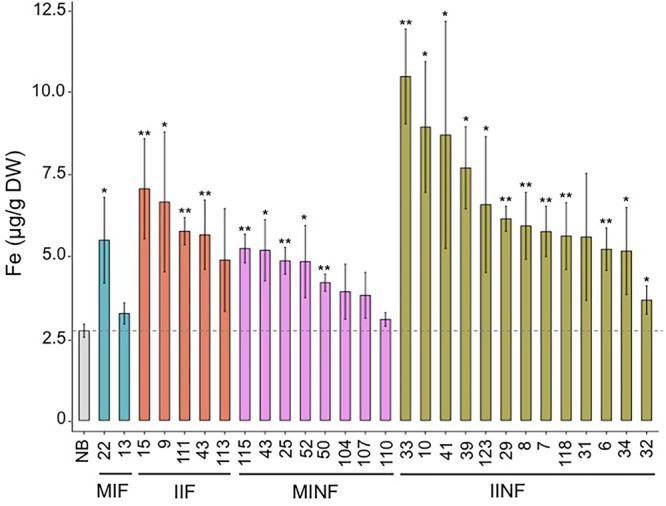
**Iron concentration in the polished T_2_ grains of lines with different promoter–transgene combinations.** Values are the average of three biological replicates (±standard deviation). Asterisks above the bars indicate significantly higher values calculated using Student’s *t*-test in comparison to the Nipponbare (NB) control (^∗^*P*<0.05, ^∗∗^*P*<0.01), respectively. NB, rice cultivar Nipponbare; for other abbreviations, see legend of **Figure 2**.

Most of the transgenic MIF, IIF, MINF, and IINF lines (see **Figure 1**) had increased iron concentrations of more than 5.0 μg g^-1^ DW in polished T_2_ grains as compared to 2.73 μg/g DW iron in the NB control (**Figure 3**). The highest iron concentration in MIF lines was 5.48 μg/g DW (MIF 22), in IIF lines 7.05 μg/g DW (IIF 15), and in MINF lines 5.23 μg/g DW (MINF 115). Several of the IINF lines had >7.0 μg/g DW iron in polished grains, which is significantly above the median of the MINF, MIF, and IIF lines. Highest iron concentration was observed in the polished grains of IINF 33 (10.46 μg/g DW), which is 3.8-fold higher than the NB control. The IINF and IIF lines generally have higher endosperm iron concentration than MINF and MIF lines, suggesting that the *AtIRT1* promoter is more effective than the *MsENOD12B* promoter in our biofortification strategy. However, a robust conclusion about promoter efficacy requires the analysis of many more transgenic lines than could be accomplished in our study. The endosperm iron concentration in the transgenic lines does not always correlate with the expression levels of *AtIRT1*, neither under the control of the *MsENOD12B* nor *AtIRT1* promoter (**Figure 2**), suggesting that other processes may influence iron accumulation in independent transgenic lines as well. But the data in **Figure 3** suggests that the concerted expression of *AtIRT1, AtNAS1*, and *PvFER* is a more effective strategy to increase endosperm iron levels as compared to the expression of *AtIRT1* and *PvFER*, irrespective of the promoters.

**FIGURE 4:**
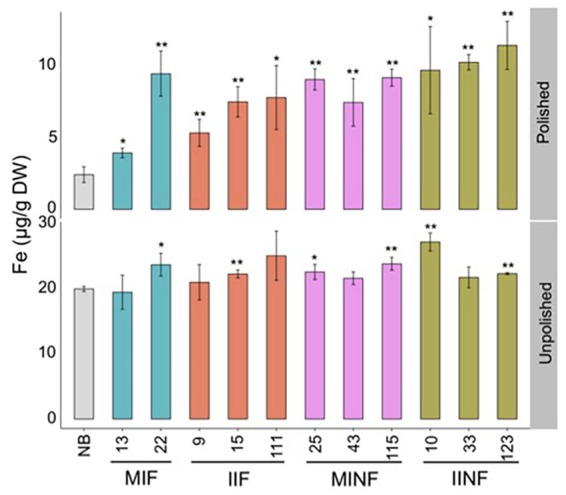
**Iron distribution in the T_3_ grains of selected transgenic lines.** Iron concentration in the T_3_ grains of transgenic plants and Nipponbare (NB) control. Values are the average of three biological replicates (±standard deviation). Asterisks above the bars indicate significantly higher values calculated using Student’s *t*-test in comparison to the NB control (^∗^*P*<0.05, ^∗∗^*P*<0.01), respectively. For abbreviations, see legend of **Figure 2**.

Analysis of selected lines confirmed that the T2 grain iron increases were maintained in both polished and unpolished T3 grains (**Figure 4**). Polished grains of MIF, IIF, MINF, and IINF had up to 3.9-, 3.2-, 3.8-, and 4.7-fold increased iron concentration as compared to the NB control, respectively. IINF T3 grains had the highest iron accumulation in the endosperm with more than 10 μg/g DW in line 123. The transgenic lines also had increased iron accumulation in the unpolished T3 grains, although the difference to the NB control was significantly lower, further substantiating the iron increase in the endosperm (**Figure 4**). Prussian blue staining of transgenic grains confirmed the increased iron localization in the endosperm (Supplementary Figure S2). Iron localization in the NB control was restricted to the radical and scutella in the embryo and to the aleurone cell layer, while in the transgenic grains iron also accumulated in the endosperm.

### The Accumulation of Other Divalent Metals is Increased in Polished Transgenic Grains

**FIGURE 5:**
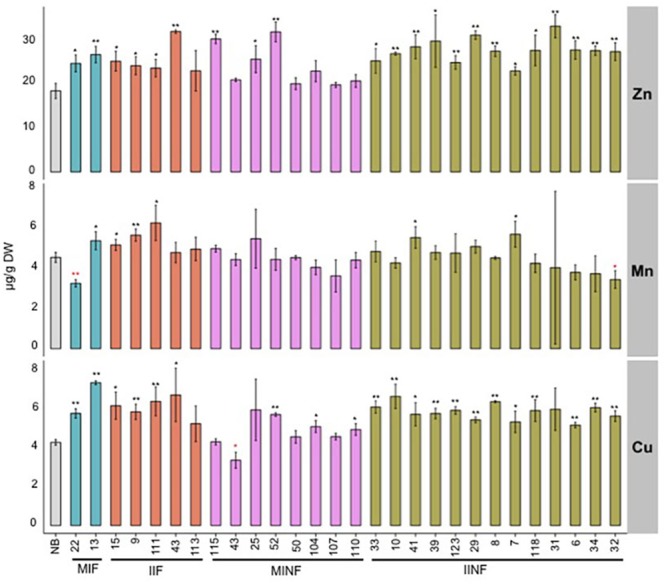
**Zinc, manganese, and copper concentrations in the polished T_2_ grains of lines expressing different promoter–transgene combinations.** Values are the average of three biological replicates (±standard deviation). Black and red asterisks above the bars indicate significantly higher or lower values calculated using Student’s *t*-test in comparison to the NB control (^∗^*P*<0.05, ^∗∗^*P*<0.01), respectively. For abbreviations, see legend of **Figure 2**.

In addition to iron, other metal ions including zinc, manganese, and copper were also quantified in the T2 transgenic grains. Zinc concentration was significantly increased in polished grains of most transgenic lines compared to the NB control (**Figure 5**). The highest zinc concentration of 33.17 μg/g DW was found in the polished grains of IINF31, which is 1.8-fold higher than the NB control. Six of the 28 transgenic lines had higher manganese concentration with up to 6.22 μg/g DW in IIF111. Two of the 28 transgenic lines had slightly reduced manganese levels, but this did not correlate with higher iron levels. Most transgenic lines also had increased copper levels in polished grains, ranging between 5.00 and 7.43 μg/g DW, as compared to 4.33 μg/g DW in NB polished grains.

### Iron Homeostasis in Shoots and Roots of the Transgenic Rice Lines Grown in Iron Sufficient and Deficient Conditions

**FIGURE 6:**
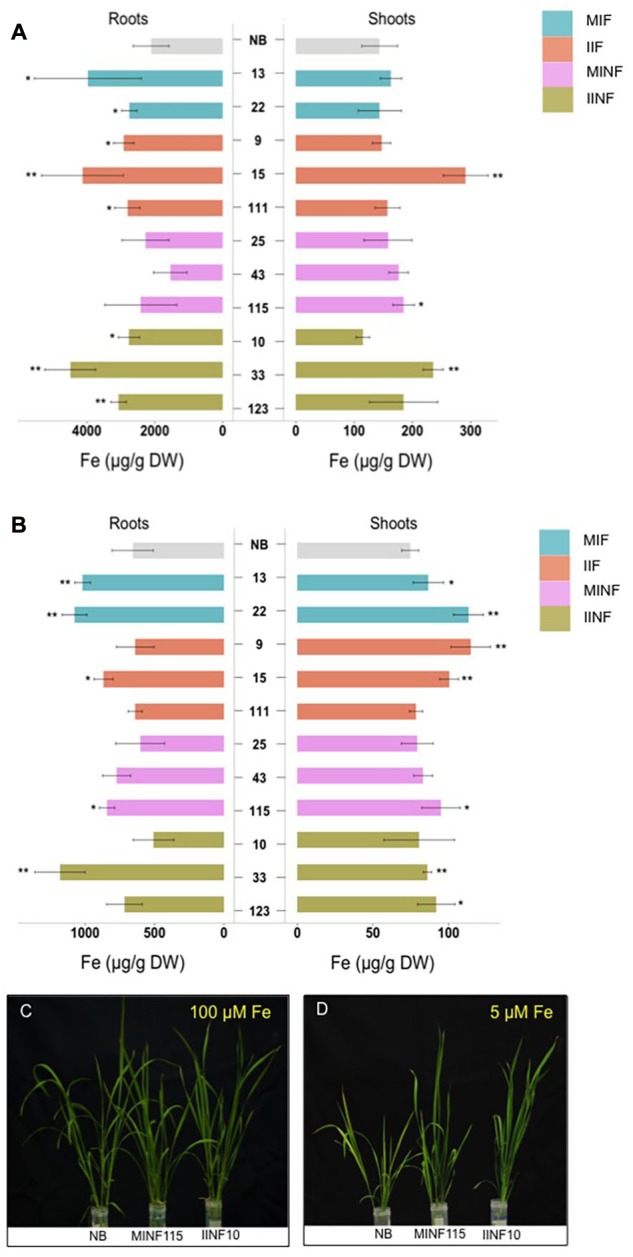
**Iron concentrations in shoots and roots of transgenic lines grown in different iron conditions.** Iron concentration in roots and shoots of seedlings that were grown in sufficient **(A)** and deficient **(B)** iron concentration conditions for 1 week. Values are the average of four biological replicates (±standard deviation). Note the differences in scale of the *x*-axis **(A,B)** indicating the measured iron concentration. Asterisks above the bars indicate significantly higher values calculated using Student’s *t*-test in comparison to the NB control (^∗^*P*<0.05, ^∗∗^*P*<0.01), respectively. Phenotypic appearance of transgenic lines and NB control after the seedlings were grown in sufficient **(C)** and deficient **(D)** iron concentration conditions for 2 weeks. For abbreviations, see legend of **Figure 2**.

Iron accumulation in the shoots and roots of the tested transgenic lines containing increased grain iron concentration was variable but in most lines less than 50% different from the NB control under iron sufficient and deficient conditions (**Figure 6**). Roots of the NB control had 2112.17 μg/g DW iron when sufficient iron was available, but only 658.16 μg/g DW iron under iron deficiency conditions. Under iron sufficient conditions, line IIF15 had a 2.1-fold increased iron concentration in the shoot while most other lines had shoot iron levels similar to the NB control (**Figure 6A**). Root iron levels under deficiency conditions were similar to NB roots in most transgenic lines except lines MIF13, MIF22, IIF15, MINF115, and IINF33, which showed variable increases in the root iron concentration (**Figure 6B**). In contrast, most transgenic lines accumulated more iron in the shoots than the NB control under iron deficiency (**Figure 6B**). Two of the transgenic lines, MINF115 and IINF10, were exposed to iron deficiency for 2 weeks for phenotypic observation, and these lines exhibited better growth and reduced chlorosis in comparison to the control (**Figures 6C,D**). Phenotypic assessment of the transgenic lines showed that most lines were similar to the NB control with respect to the plant height, tiller numbers, days to flowering, panicle numbers and 1,000 grain weight (Supplementary Table S1).

## Discussion

We have demonstrated that tissue-specific expression of IRT1 in combination with NAS and FERRITIN from a single gene cassette can be used to increase iron levels, and lines with iron concentrations of up to 10.46 μg/g DW in the polished rice grains are reported. Conventional breeding and agronomic micronutrient fortification have shown promises for improving zinc but not iron concentration in cereal grains (Cakmak, 2008; Ahmad et al., 2012; Phattarakul et al., 2012). Among the genetic engineering strategies, those combining two or more genes were relatively more successful in increasing rice endosperm iron concentrations (Wirth et al., 2009; Masuda et al., 2012, 2013; Aung et al., 2013). For example, we previously reported that endosperm-specific expression of *FERRITIN* and *PHYTASE* together with the constitutive expression of *NAS* (NFP rice) resulted in a sixfold increased rice endosperm iron concentration (Wirth et al., 2009). A recent study reported 15 μg/g DW iron in polished grains of a field-grown transgenic line expressing *NAS* and *FERRITIN* (Trijatmiko et al., 2016), but the reported lines have two transgene cassette copies integrated as an inverted repeat. There is currently little evidence that the duplicated and inverted transgene locus in these lines can be stably maintained over generations. It has been repeatedly reported by different laboratories that two or more insertions of a transgene often result in epigenetic transgene silencing in later plant generations (e.g., Kumpatla and Hall, 1998; Li et al., 2002; Tang et al., 2006; Rajeevkumar et al., 2015). Therefore, it is likely that transgene cassette copies integrated as an inverted repeat may not inherit the iron and zinc trait over multiple generations. This makes the suitability of such an inverted transgene cassette repeat line questionable for breeding programs. Therefore, development of iron-biofortified rice lines should be continued using best single copy transgene cassette insertion lines with Fe accumulation that is stably inherited and that can be bred efficiently and robustly into widely grown rice cultivars. Here we report single copy cassette insertion rice lines with endosperm Fe concentrations reaching 10.46 μg/g (nearly 70% of the 15 ppm recommended by Bouis et al., 2011) in the polished rice grains, which is a significant increase as compared to many of the previously reported lines.

We recently reported that transformation of NFP rice (engineered for higher grain iron) with *AtIRT1* could further increase the endosperm iron concentration by twofold, producing lines containing 9.67 μg/g DW iron in polished grains (Boonyaves et al., 2016). This encouraged us to engineer rice lines expressing *AtIRT1, AtNAS1*, and *PvFERRITIN* from a gene cassette present in the transgenic lines as a single locus. Many previous studies, including our own, have shown that single transgenic loci are robustly maintained over several generations. This approach minimizes the risk of transgenes segregating in consecutive generations. Importantly, iron levels in polished grains of the transgenic lines expressing *AtIRT1, AtNAS1*, and *PvFERRITIN* from a single cassette reported here are similar or higher than in NFP lines transformed with *AtIRT1* (Boonyaves et al., 2016), suggesting that combining the three genes in a single locus has no negative consequences on their performance or significantly changes iron homeostasis in the plants. Furthermore, our results show that the concerted activity of IRT, NAS, and FERRITIN was superior for increasing endosperm iron content to combining IRT and FERRITIN only. Importantly, the transformed lines performed at par with the non-transformed cultivar in terms of several recorded growth parameters.

The expression of NAS increases NA and DMA production, which facilitates efficient uptake and long distance transport of iron (Hell and Stephan, 2003; Inoue et al., 2003; Takahashi et al., 2003; Kobayashi and Nishizawa, 2012). Chelators that facilitate translocation of iron are often also involved in uptake and transport of other metal ions. For example, in addition to iron, PS and NA transport other essential metal ions including zinc, manganese and copper (Haydon and Cobbett, 2007). Enzymes involved in PS biosynthesis are often upregulated under both iron, zinc, or copper deficiency conditions (Wintz et al., 2003; Suzuki et al., 2006). It is therefore not unexpected that expression of homologous or heterologous *NAS* genes in rice lines increases both iron and zinc content in the grains. In most of the rice plants engineered for higher endosperm iron, increases of iron were accompanied by moderate increases of zinc in the grains (Vasconcelos et al., 2003; Lee et al., 2009b; Masuda et al., 2009, 2012; Wirth et al., 2009; Johnson et al., 2011; Zhang et al., 2012; Tan et al., 2015; Trijatmiko et al., 2016), indicating that transgenic iron biofortification strategies have a positive effect on zinc levels. Expression of *IRT1* in rice also increased zinc concentration in rice grains (Lee and An, 2009; Tan et al., 2015), which can be expected from the metal ion transport capacity of the IRT1 transporter (Korshunova et al., 1999). We found increases for zinc, copper, and manganese in polished grains of the transgenic rice lines expressing the *AtIRT1, AtNAS1*, and *PvFERRITIN* gene cassette, but increases in iron concentration were most significant. The greenhouse performance of the transgenic lines with regard to micronutrient biofortification and agronomic performance is a promising proof-of-concept that nutritionally sufficient iron levels in polished rice grains can be achieved. Field testing and breeding into consumer-preferred varieties is now necessary to demonstrate the agronomic robustness and nutritional benefit of the high-iron trait.

## Author Contributions

NB and WG conceived and designed the study, KB and T-YW carried out the experiments and analyzed the data, KB and NB wrote the manuscript, WG and NB edited the manuscript. All authors have read and approved the final manuscript.

## Conflict of Interest Statement

The authors declare that the research was conducted in the absence of any commercial or financial relationships that could be construed as a potential conflict of interest.

## Footnotes

1 https://www-s.nist.gov/srmors/view_detail.cfm?srm=1568a

2 https://genevestigator.com/gv/
