# Supplementary material for: Enhanced Grain Iron Levels in Rice Expressing an IRON-REGULATED METAL TRANSPORTER, NICOTIANAMINE SYNTHASE, and FERRITIN Gene Cassette
Source: Front Plant Sci. 2017 Feb 7;8:130. doi: 10.3389/fpls.2017.00130 (PMC5293767; doi:10.3389/fpls.2017.00130)
Supplement: Supplementary file 1 [file Table_1.PDF]

Supplementary table 1 Phenotypic parameters of transgenic lines expressing *AtIRT1* and *PvFER* genes (MIF and IIF); and *AtIRT1, AtNAS1* and *PvFER* genes (MINF and IINF)

Plant line	Days to flowering	Plant height (cm)	Tiller number	Panicle number	1000GW*
Nipponbare	66.0 ± 0.0	52.3 ± 1.6	12.7 ± 2.5	4.0 ± 1.7	24.1± 0.8
MIF13	67.0 ± 0.0	60.3 ± 1.5	6.7 ± 2.5	4.3 ± 1.2	23.4 ± 0.5
MIF22	67.3 ± 0.6	64.3 ± 2.2	17.7 ± 2.5	11.7 ± 1.2	24.0 ± 1.2
IIF9	56.0 ± 0.0	53.1 ± 2.8	17.0 ± 1.7	11.3 ± 4.0	23.6 ± 0.5
IIF15	68.0 ± 2.0	65.8 ± 3.5	16.0 ± 0.0	8.3 ± 0.6	24.0 ± 0.5
IIF43	61.7 ± 2.3	50.5 ± 4.0	17.3 ± 6.7	10.7 ± 4.6	20.0 ± 0.5
IIF111	70.0 ± 0.0	66.6 ± 0.4	13.7 ± 2.1	5.7 ± 1.2	20.2 ± 0.5
IIF113	56.0 ± 0.0	42.6 ± 15.2	20.7 ± 2.5	11.3 ± 3.1	20.0 ± 0.5
MINF25	68.0 ± 1.7	46.3 ± 4.0	14.3 ± 7.8	7.3 ± 2.1	22.8 ± 1.1
MINF43	64.0 ± 1.7	46.0 ± 4.6	13.3 ± 0.6	10.7 ± 0.6	26.8 ± 0.4
MINF50	63.0 ± 0.0	56.8 ± 1.1	21.0 ± 5.7	19.5 ± 3.5	27.0 ± 2.1
MINF52	56.3 ± 0.6	51.0 ± 1.2	20.0 ± 5.6	17.7 ± 5.5	ND
MINF104	66.0 ± 3.0	57.3 ± 2.5	14.3 ± 3.1	7.7 ± 3.1	ND
MINF107	64.7 ± 1.2	53.9 ± 1.1	18.0 ± 3.0	7.0 ± 1.0	ND
MINF110	66.3 ± 2.5	52.0 ± 5.8	14.7 ± 3.5	10.3 ± 3.5	ND
MINF115	57.0 ± 0.0	51.3 ± 1.6	20.7 ± 7.0	12.0 ± 5.3	25.3 ± 0.4
IINF6	62.7 ± 2.3	53.5 ± 2.3	22.0 ± 2.7	14.7 ± 3.1	ND
IINF7	61.0 ± 2.6	46.3 ± 8.6	20.0 ± 7.0	9.0 ± 3.6	18.7 ± 3.4
IINF8	61.0 ± 1.7	51.8 ± 1.5	18.0 ± 2.6	11.3 ± 1.5	ND
IINF10	63.0 ± 0.0	54.1 ± 2.3	20.3 ± 2.3	13.0 ± 1.7	23.5 ± 0.7
IINF29	58.0 ± 1.7	47.6 ± 2.6	20.7 ± 4.5	14.0 ± 3.6	ND
IINF31	61.0 ± 1.73	45.6 ± 3.0	17.7 ± 8.5	10.7 ± 6.8	ND
IINF32	60.7 ± 2.1	48.9 ± 1.6	19.3 ± 1.5	10.7 ± 0.6	ND
IINF33	76.0 ± 1.7	46.7 ± 4.5	17.0 ± 1.7	6.3 ± 1.1	23.2 ± 0.8
IINF34	69.3 ± 7.8	45.5 ± 3.8	17.7 ± 9.1	11.0 ± 7.9	23.8 ± 0.4
IINF39	64.3 ± 1.2	56.6 ± 1.4	20.0 ± 0.0	14.7 ± 2.5	ND
IINF41	66.0 ± 0.0	50.0 ± 3.0	19.0 ± 1.0	6.0 ± 0.0	ND
IINF118	67.7 ± 8.1	46.9 ± 15.8	19.0 ± 7.5	7.0 ± 4.0	ND
IINF123	66.3 ± 3.2	45.3 ± 1.0	17.3 ± 4.7	6.3 ± 2.1	22.8 ± 0.4

* ND. No data available.
