# Supplementary material for: Enhanced Grain Iron Levels in Rice Expressing an IRON-REGULATED METAL TRANSPORTER, NICOTIANAMINE SYNTHASE, and FERRITIN Gene Cassette
Source: Front Plant Sci. 2017 Feb 7;8:130. doi: 10.3389/fpls.2017.00130 (PMC5293767; doi:10.3389/fpls.2017.00130)
Supplement: Supplementary file 2 [file Table_2.PDF]

Supplementary table 2 Primer sequences used for quantitative analysis of transgenes expression

Nr.	Gene	Accession ID	Forward primer	Reverse primer
**1**	*AtIRT1*	NM_118089	tcgaaggcatgggtcttg	acgccataacaaatttcttcatatt
**2**	*AtNAS1*	AY072364	gcacttggagaaacacatgg	tctgagagcatgagcactcc
**3**	*PvFERRITIN*	X58274	ccgatcaagaatgtaccctca	aattccattgcatataacgcatc
**4**	*Os01g0147200*	NM_001048546	agcagctgaaagcaccaaa	cacgcccttcaacactgag
