# Supplementary material for: Enhanced Grain Iron Levels in Rice Expressing an IRON-REGULATED METAL TRANSPORTER, NICOTIANAMINE SYNTHASE, and FERRITIN Gene Cassette
Source: Front Plant Sci. 2017 Feb 7;8:130. doi: 10.3389/fpls.2017.00130 (PMC5293767; doi:10.3389/fpls.2017.00130)
Supplement: Supplementary file 3 [file Image_1.PDF]

Supplementary material

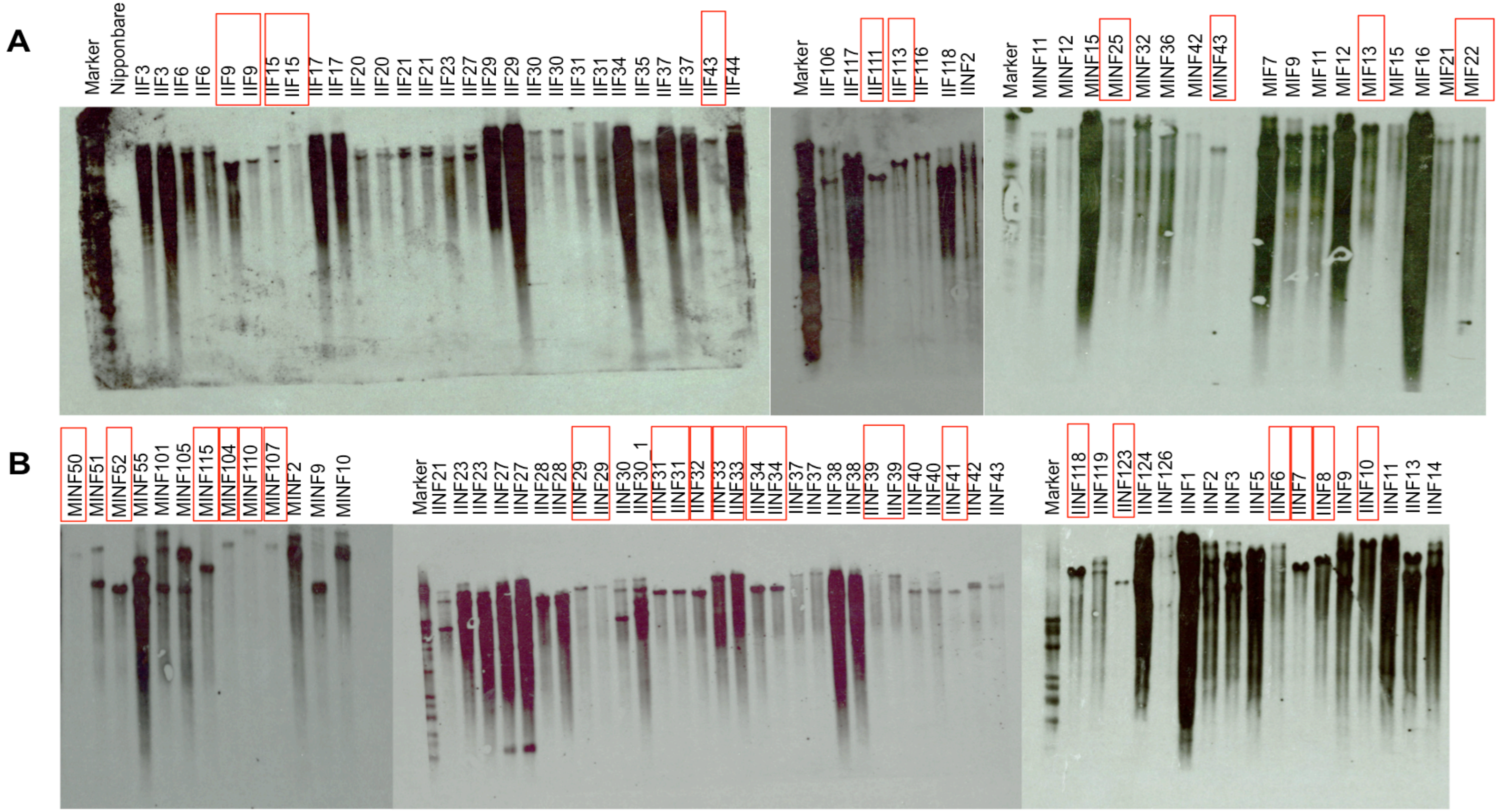

Supplementary figure 1 Southern hybridization analysis in transgenic lines expressing *AtIRT1* and *PvFER* genes; and *AtIRT1, AtNAS1* and *PvFER* genes. The transgenic copy number in the independent transgenic lines was detected by *AtIRT1* **(A)** or *AtNAS1* **(B)** probe. The transgenic lines that contained single copy of transgene insertion were selected (marked by boxes).
