# Supplementary material for: Enhanced Grain Iron Levels in Rice Expressing an IRON-REGULATED METAL TRANSPORTER, NICOTIANAMINE SYNTHASE, and FERRITIN Gene Cassette
Source: Front Plant Sci. 2017 Feb 7;8:130. doi: 10.3389/fpls.2017.00130 (PMC5293767; doi:10.3389/fpls.2017.00130)
Supplement: Supplementary file 4 [file Image_2.PDF]

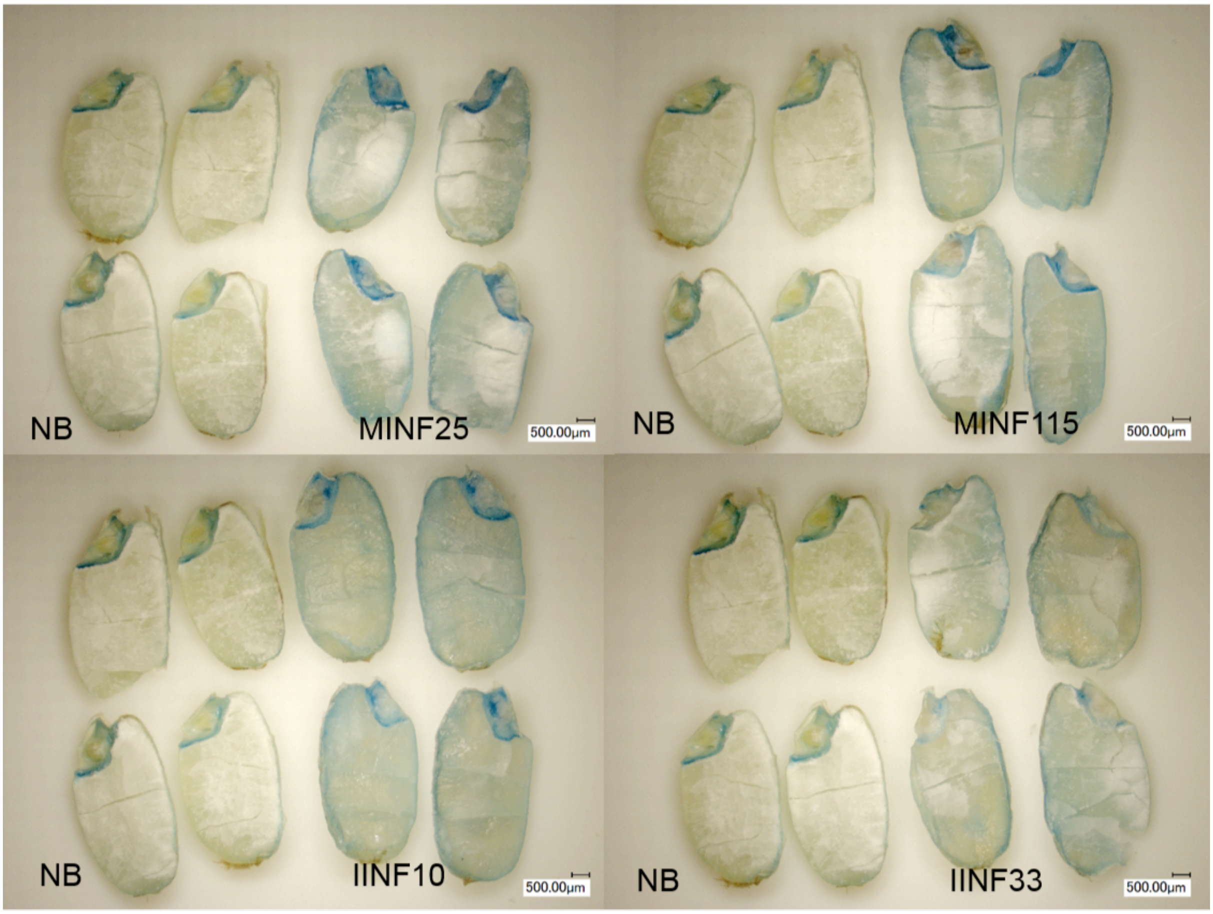

Supplementary figure 2. Iron localization in the unpolished T_3 grains of selected transgenic lines and NB control. Unpolished grains were cut longitudinally prior to Prussian blue staining.
